# Synthetic quantitative MRI through relaxometry modelling

**DOI:** 10.1002/nbm.3658

**Published:** 2016-10-18

**Authors:** Martina F. Callaghan, Siawoosh Mohammadi, Nikolaus Weiskopf

**Affiliations:** ^1^ Wellcome Trust Centre for NeuroimagingUCL Institute of Neurology, University College LondonLondonUK; ^2^ Department of Systems NeuroscienceUniversity Medical Center Hamburg‐EppendorfHamburgGermany; ^3^ Department of NeurophysicsMax Planck Institute for Human Cognitive and Brain SciencesLeipzigGermany

**Keywords:** magnetization transfer, relaxometry, synthetic quantitative MRI

## Abstract

Quantitative MRI (qMRI) provides standardized measures of specific physical parameters that are sensitive to the underlying tissue microstructure and are a first step towards achieving maps of biologically relevant metrics through *in vivo* histology using MRI. Recently proposed models have described the interdependence of qMRI parameters. Combining such models with the concept of image synthesis points towards a novel approach to synthetic qMRI, in which maps of fundamentally different physical properties are constructed through the use of biophysical models. In this study, the utility of synthetic qMRI is investigated within the context of a recently proposed linear relaxometry model. Two neuroimaging applications are considered. In the first, artefact‐free quantitative maps are synthesized from motion‐corrupted data by exploiting the over‐determined nature of the relaxometry model and the fact that the artefact is inconsistent across the data. In the second application, a map of magnetization transfer (MT) saturation is synthesized without the need to acquire an MT‐weighted volume, which directly leads to a reduction in the specific absorption rate of the acquisition. This feature would be particularly important for ultra‐high field applications. The synthetic MT map is shown to provide improved segmentation of deep grey matter structures, relative to segmentation using *T*_1_‐weighted images or *R*_1_ maps. The proposed approach of synthetic qMRI shows promise for maximizing the extraction of high quality information related to tissue microstructure from qMRI protocols and furthering our understanding of the interrelation of these qMRI parameters.

**Abbreviations Used**
EPI: echo planar imaging
FLASH: fast low angle shot
GM: grey matter
MNI: Montreal Neurological Institute
MPM: multi‐parameter mapping
MT: magnetization transfer
MTR: magnetization transfer ratio
PD: proton density
qMRI: quantitative MRI
*R*_1_: longitudinal relaxation rate
*R*_2_*: effective transverse relaxation rate
SAR: specific absorption rate
SPM: Statistical Parametric Mapping
TPM: tissue probability map
VBM: voxel‐based morphometry
WM: white matter
WTCN: Wellcome Trust Centre for Neuroimaging

## 1 INTRODUCTION

Quantitative MRI (qMRI) provides standardized measures of specific physical parameters that are sensitive to the underlying tissue microstructure. The standardized nature of these parameters facilitates comparison across sites and time points, which greatly improves the sensitivity and efficiency of multi‐centre and longitudinal studies.^1^ qMRI is also the first step towards achieving maps of biologically relevant metrics through *in vivo* histology using MRI.^2^ However, since qMRI metrics are sensitive to multiple biological factors (e.g. fibre density, water, myelin and iron content), multiple parameters are needed to improve biological specificity.^3^ The multi‐parameter mapping (MPM) protocol^1^ is one such quantitative imaging approach, in which data are combined to calculate maps of the longitudinal relaxation rate (*R*_1_), the effective transverse relaxation rate (*R*_2_*), the magnetization transfer saturation (MT) and the effective proton density (PD*).

Given a set of qMRI parameters, such as are generated with the MPM protocol, it is possible to synthesize images with arbitrary contrast weighting through use of the appropriate MRI signal model.^4^, ^5^, ^6^ This provides a flexible and time efficient approach to investigating tissue integrity and pathology, e.g. by generating multiple inversion recovery images covering a range of inversion times. Such a synthetic approach has been proposed as a first step towards the adoption of fully quantitative imaging within a clinical environment,^7^ and clinical utility has been demonstrated, e.g. in the visualization of tumours.^8^

Going beyond physical models that describe the MRI signal as a function of scanner parameters, such as flip angle, repetition and echo times, biophysical models that describe the interdependence of MRI parameters, such as *R*_1_, proton density (PD) and macromolecular tissue volume fraction, have more recently been proposed.^9^, ^10^, ^11^ Combining such models with the concept of synthesizing images points towards an alternative approach: synthetic qMRI. In this case quantitative maps of fundamentally different physical properties are constructed through the use of biophysical models as distinct from constructing simple weighted images as in the conventional approach to synthetic MRI. Such modelling approaches may enhance the robustness of quantitative imaging protocols that aim to quantify multiple parameters. For example, high resolution (finer than 1 mm isotropic) and whole brain coverage leads to extended MPM protocol durations (25 min or more) and therefore increased vulnerability to motion, which could render valuable data unusable. In addition, at ultra‐high field (>3 T) acquiring an MT‐weighted volume can be particularly challenging due to the supra‐linear increase in specific absorption rate (SAR) with field strength. The absence of an MT‐weighted acquisition, due to either motion or SAR limitations, is particularly problematic since it prohibits the construction of an MT map, yet these have been shown to facilitate improved segmentation of deep grey matter (GM) structures.^12^ These segmentation benefits are of great clinical importance because changes in regions such as the basal ganglia are associated with a number of pathological conditions,^13^ including Parkinson's and Huntington's diseases, both of which are associated with involuntary movement such that remaining still during data acquisition may be particularly difficult for these patient groups.

In this study, the utility of *synthetic qMRI* is investigated within the context of the recently proposed linear relaxometry model.^11^ In this model, which stems from the fundamental principles of the fast exchange regime,^14^ the components of the apparent longitudinal relaxation rate (*R*_1_) are expressed as a weighted sum of other qMRI metrics. Quantitative maps of MT and effective transverse relaxation rate (*R*_2_*) are used as surrogates for the macromolecular and paramagnetic contributions to *R*_1_ respectively. This model can be constructed on a participant‐specific basis by pooling over GM and white matter (WM). The coefficients of this general linear model, which are global scalars for the whole brain, exhibited remarkable stability across a large, heterogeneous cohort.^11^ This stability indicates that the mean of the population‐derived model coefficients could be used on newly acquired maps to achieve the goal of synthesizing a full set of quantitative parameter maps from just a sub‐set of the MPM protocol. The utility of doing so is demonstrated with two applications in the neuroimaging domain. In the first application, artefact‐free quantitative maps are synthesized from motion‐corrupted data by exploiting the over‐determined nature of the relaxometry model and the fact that the artefact is inconsistent across the quantitative maps, and is instead captured by the residuals of the model. In the second application, an MT map is synthesized without the use of an MT‐weighted volume, which directly leads to a reduction in the SAR of the protocol. Using the synthesized MT map, we assess whether the previously established improvement in segmenting deep GM structures, relative to segmentation using *T*_1_‐weighted images, is maintained.

## 2 THEORY: LINEAR RELAXOMETRY MODEL

In the absence of exogenous contrast agents, the measured *R*_1_ is dominated by contributions from free water spins, bound water spins at macromolecular sites and a smaller, spatially varying contribution from iron sites.^15^, ^16^ Under conditions of fast exchange, the measured *R*_1_ can be expressed as a weighted sum of the relaxivities of these compartments^14^:

(1) $$R_{1}=R_{1f}+f_{M}r_{1M}+f_{\mathrm{FE}}r_{1\mathrm{FE}}+\sum_{j}f_{j}r_{1j}.$$

Here *R*_1f_ is the relaxation rate of free water; *f*_M_ is the fraction of spins at macromolecular sites with relaxivity *r*_1M_; *f*_FE_ is the fraction of spins at iron sites with relaxivity *r*_1FE_; the index *j* sums over any unspecified contributions. The relaxivity describes the increase in the relaxation rate relative to free water sites, e.g. *r*_1M_ = *R*_1M_ − *R*_1f_, where *R*_1M_ is the relaxation rate at macromolecular sites. A model of the apparent *R*_1_ purely based on imaging data can be constructed by replacing the known contributors to *R*_1_ with voxel‐wise surrogate imaging markers^11^:

(2) $$R_{1}\left(r\right)=\beta_{0}+\beta_{1}\mathrm{MT}\left(r\right)+\beta_{2}R_{2}^{*}\left(r\right)+\varepsilon \left(r\right).$$

Here, *R*_1f_ is taken to be a constant, *β*_0_. The macromolecular term, *f*_M_ *r*_1M_, is replaced by a map of MT saturation^17^; *r* in parentheses denotes spatial location, indicating the voxel‐wise nature of the model. A single, global model coefficient, *β*_1_, which holds for both GM and WM, describes the macromolecular contribution to the measured *R*_1_. Similarly, an *R*_2_* map and additional global model coefficient, *β*_2_, are used as a surrogate for the contribution from iron sites, i.e. *f*_FE_ *r*_1FE_. *ε*(*r*) is a map of model residuals encompassing any potential unspecified contributions to *R*_1_ and noise.

A single set of *β* parameters has been shown to be sufficient to model the contributions to *R*_1_ across GM and WM.^11^ Given these model coefficients, which may either be published values or values calculated directly for the participant, a synthetic *R*_1_ map is calculated by rewriting Equation (2) as follows:

(3) $${\hat{R}}_{1}\left(r\right)=\beta_{0}+\beta_{1}\mathrm{MT}\left(r\right)+\beta_{2}R_{2}^{*}\left(r\right).$$

Similarly, a synthetic MT map is calculated by rearranging Equation (3) and using the measured *R*_1_ map:

(4) $$\hat{\mathrm{MT}}\left(r\right)=\frac{\left(\;R_{1}\left(r\right)-\beta_{0}-\beta_{2}R_{2}^{*}\left(r\right)\;\right)}{\beta_{1}}.$$

Equation (4) allows a map of MT saturation to be calculated without the acquisition of an MT‐weighted volume, since its calculation relies only on the model coefficients and the measured *R*_1_ and *R*_2_* maps.

Note also that the synthesized quantitative maps, $\;\hat{R_{1}}\left(r\right)$ and $\hat{\;\mathrm{MT}}\left(r\right)$, do not contain model residuals. This means that noise sources, such as motion artefact, that lead to inconsistencies across the constituent maps are removed. This is the proposed mechanism for motion artefact removal. As part of the MPM protocol, three contrast weightings are acquired with predominantly PD, *T*_1_ or MT weighting. The *R*_2_* map is derived solely from the decay of the signal across the echoes of the PD weighted volume whereas the *R*_1_ map relies on the combination of the PD and *T*_1_ weighted volumes. A map of MT saturation requires data from all three weighted volumes since, unlike magnetization transfer ratio (MTR) maps, it is corrected for spatially varying *T*_1_ times. Given the over‐determined nature of the model when the model coefficients are known, artefact‐free maps can be calculated using Equations (3) and (4), depending on which of the weighted volumes have been corrupted by motion according to the following scenarios.

1. The MT‐weighted acquisition is corrupted by motion. In this case, Equation (4) can be used to construct a synthetic MT map free of motion artefact. This is the optimum case for correction since the MT‐weighted acquisition contributes only to the calculation of the MT map.
2. The *T*_1_‐weighted acquisition is corrupted by motion. In this case, the *R*_1_ map will be most heavily degraded by motion artefact and Equation (3) can be used to construct a synthetic *R*_1_ with reduced artefact level.
3. The PD‐weighted acquisition or both the *T*_1_‐ and MT‐weighted volumes are corrupted by motion. In these cases, all maps will be corrupted by motion to some degree and the possibility of improving the quality of the maps is reduced. The correction achievable will depend on the extent of motion artefact across the constituent volumes.

## 3 METHODS

All data were acquired on a 3 T whole body MR system (TIM Trio, Siemens Healthcare, Erlangen, Germany) equipped with an RF body coil for transmission and a 32 channel RF head coil for receiving. The studies were approved by the local ethics committee and informed written consent was obtained from all participants prior to scanning.

### 3.1 Data acquisition

Two studies were performed to assess the utility of synthesizing quantitative parameter maps using the linear relaxometry model and maps derived from the MPM protocol. The first study assessed the performance of generating synthetic quantitative data free of motion artefact from motion‐corrupted data. The second study investigated tissue segmentation performance in a group of volunteers representing the typical population at a cognitive neuroimaging centre. Both studies utilized a whole‐brain MPM protocol with 1 mm isotropic resolution, which consisted of three spoiled multi‐echo 3D fast low angle shot (FLASH) acquisitions acquired with predominantly PD, *T*_1_ or MT weighting, as determined by the repetition time and flip angle (respectively 23.7 ms and 6° for the PD‐ and MT‐weighted acquisitions; and 18.7 ms and 20° for the *T*_1_‐weighted acquisition). For the MT‐weighted acquisition, a Gaussian RF pulse (4 ms duration, 220° nominal flip angle) was applied 2 kHz off‐resonance prior to non‐selective excitation. Gradient echoes were acquired with alternating readout gradient polarity at six equidistant echo times between 2.2 ms and 14.7 ms. Two additional echoes were acquired for the PD‐weighted acquisition at 17.2 ms and 19.7 ms. To accelerate data acquisition, parallel imaging (speedup factor of 2) was used in the anterior–posterior phase‐encoded direction and reconstructed using the GRAPPA algorithm. A partial Fourier acquisition (6/8 sampling factor) was used in the left–right phase‐encoded direction. The duration of the PD‐ and MT‐weighted acquisitions was just under 7 min. The *T*_1_‐weighted acquisition was just over 5 min. 3D echo planar imaging (EPI) data of spin and stimulated echoes with 11 different nominal flip angles ranging from 65° to 115° in 5° steps were acquired with 4 mm isotropic resolution in order to map the transmit field inhomogeneity (*T*_E_/*T*_M_/*T*_R_ = 37.06/31.20/500 ms; see also Reference^18^). Given that an EPI readout was used to acquire these data and is affected by off‐resonance effects, *B*_0_ field mapping data derived from the phase difference of a dual gradient echo acquisition were also acquired to correct for geometric distortions.^19^ A spatial map of the actual flip angle achieved was calculated by taking the inverse cosine of the ratio of the stimulated echo and spin echo images for each pair. The transmit efficiency was then calculated as the ratio of this achieved flip angle relative to the nominal flip angle. In areas of excessively high *B*_0_ off‐resonance, the flip angle estimates were interpolated to avoid bias. This efficiency was used to spatially correct the nominal flip angle when computing the quantitative maps.^18^, ^20^ The scan time for the full MPM protocol was approximately 24 min.

Quantitative maps were derived from the MPM protocol in the Statistical Parametric Mapping framework (SPM12.0, Wellcome Trust Centre for Neuroimaging (WTCN), London) using bespoke MATLAB tools (MathWorks, Natick, MA, USA). Briefly, regression of the log signal from the eight PD‐weighted echoes was used to calculate a map of *R*_2_*. The first six echoes for each contrast weighting were then averaged to increase the signal‐to‐noise ratio.^21^ Quantitative maps of the apparent *R*_1_ were calculated from the PD‐ and *T*_1_‐weighted volumes using the rational approximation of the Ernst equation^22^ incorporating correction for transmit field efficiency as described above.^18^ The FLASH acquisitions use both RF (50° phase increment) and gradient spoiling to minimize unwanted magnetization coherence pathways. However, residual errors can remain. To address this, we simulated the FLASH acquisitions using Bloch‐Torrey equations for a range of expected transmit field efficiency. The correction parameters describing the linear dependence of the actual *T*_1_ value on the apparent *T*_1_ were derived from these simulations as described in Reference 23 and used to correct for imperfect spoiling of transverse magnetization. Semi‐quantitative maps of the percentage loss of magnetization resulting from the pre‐pulse in the MT‐weighted acquisition were calculated as described by Helms et al.^17^ accounting for spatially varying *T*_1_ times and flip angle inhomogeneities.^1^

### 3.2 Motion artefact correction study

MT and *R*_1_ maps were synthesized for 12 motion‐affected datasets. These datasets had been excluded from various neuroimaging studies on the basis of failing visual inspection because of excessive levels of artefact consistent with intra‐scan motion in one or more of the constituent FLASH volumes. Equations (3) and (4) were used to generate synthetic maps of *R*_1_ and MT respectively using the mean relaxometry model coefficients reported in reference 11. The success of motion artefact removal was evaluated by expert raters (*n* = 5, experienced physicists from WTCN), given that such evaluation has been shown to be a robust means of assessing motion artefact correction.^24^, ^25^, ^26^ Each rater was presented with the original map and the corresponding synthetic map and had to decide which image had the least motion artefact, i.e. performed a forced choice rating assessment. The evaluation was carried out by each rater in two blocks, one for MT maps and one for *R*_1_ maps. For a given parameter, the original and synthetic maps were presented together, with identical windowing. The relative position of the maps was randomized across cases. The expert raters were free to navigate through the maps in all three planes and given as much time as required to decide which map had the least motion artefact. For each of the 12 cases, a binomial distribution was used to test the null hypothesis that, given five raters, the probability of selecting the synthesized map did not significantly differ from chance, i.e. 50% for this forced choice assessment. The threshold for significance was *p* < 0.05.

### 3.3 Voxel‐based morphometry (VBM) group study

MPM data were acquired from a group of 30 healthy volunteers (13 male, age range 18–25 years, mean 21.6 years, std dev. 1.9 years). *R*_1_ and *R*_2_* maps were used in Equation (4) to generate synthetic maps of MT, again using the mean model coefficients reported in Reference 11.

For each participant, the first echo of the *T*_1_‐weighted FLASH volume (*T*_E_ = 2.2 ms), the *R*_1_ map, the measured MT map and the synthetic MT map were segmented into GM and WM probability maps using the unified segmentation approach^27^ as implemented in SPM12.0. Default settings were used with one exception. The segmentation routine estimates the signal modulation imposed by the net sensitivity field of the receiving RF coil. This estimation is regularized with an *a priori* estimate of the sensitivity field. Given that the quantitative maps were not modulated by this receive sensitivity field, the regularization was increased (from default to ‘very heavy regularization’) when segmenting quantitative maps.

To achieve optimal inter‐subject registration, the tissue probability maps (TPMs) derived from the MT maps were used to spatially normalize the data using the non‐linear diffeomorphic DARTEL algorithm^28^ as implemented in SPM12.0. The resulting DARTEL template and deformation fields were used to normalize the GM TPMs to the Montreal Neurological Institute (MNI) stereotactic space. The probability maps were scaled by the Jacobian determinants of the deformation field as recommended for VBM studies^29^ and smoothed with an isotropic Gaussian kernel of 3 mm full width at half maximum while preserving the 1 mm isotropic voxel size in MNI space.

A voxel‐wise two‐tailed paired *t* test was carried out within SPM12 to assess GM volume differences between the synthetic MT map and each of the other image types. Significance was defined as voxels having a *p* value less than 0.05 after small volume and family‐wise error corrections for multiple comparisons. An explicit mask described by Callaghan et al.^30^ was used to exclude cerebrospinal fluid voxels. Given the *a priori* hypothesis that GM tissue classification would be variable for deep GM structures, the statistical analysis was restricted to a central search volume focusing on these structures. A sphere with a radius of 4 cm, centred on the basal ganglia, was used.

## 4 RESULTS

### 4.1 Motion artefact correction

**Figure 1:**
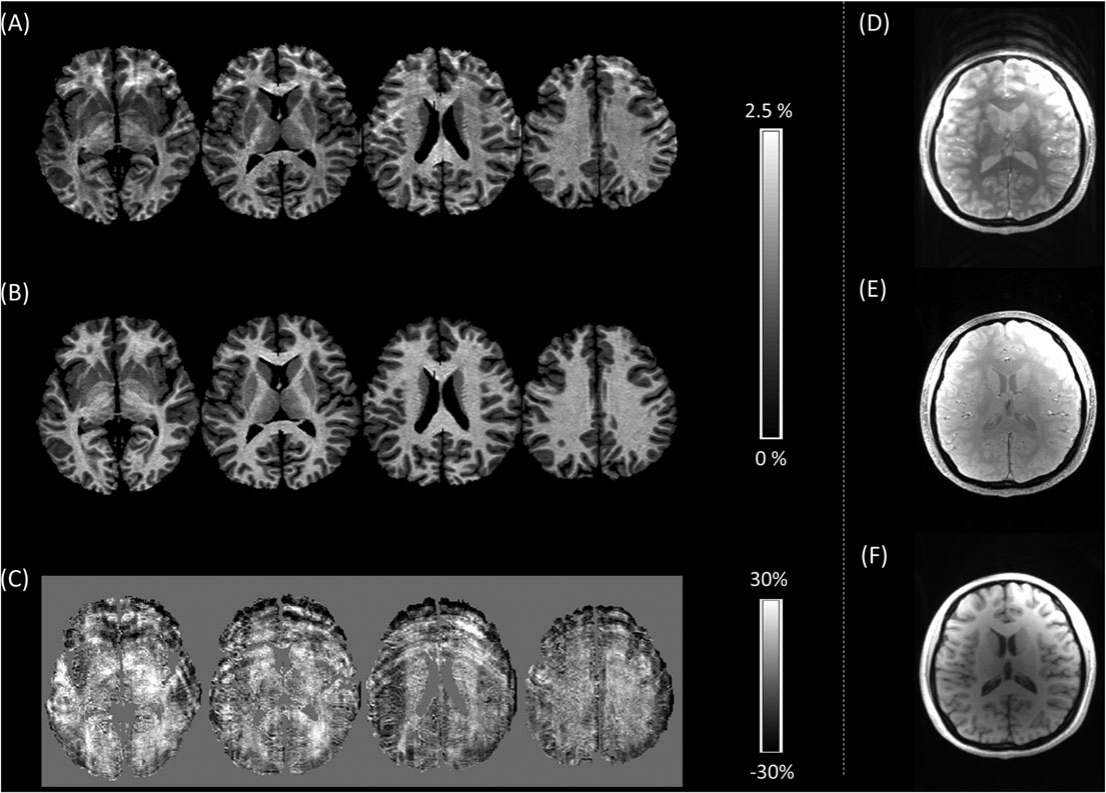
A, Multiple axial slices through the motion‐corrupted MT map of Participant 1; B, the synthetic MT map generated using the linear relaxometry model; C, the model residuals, expressed as the percentage of the mean of the measured and synthetic MT values, which capture the motion artefact. D‐F, A single slice through the weighted volumes shows that the MT‐weighted acquisition D, has been corrupted by motion whereas the PD‐ E, and *T*_1_‐weighted F, acquisitions are free of motion artefact. This is the optimal scenario for motion artefact correction

**Figure 2:**
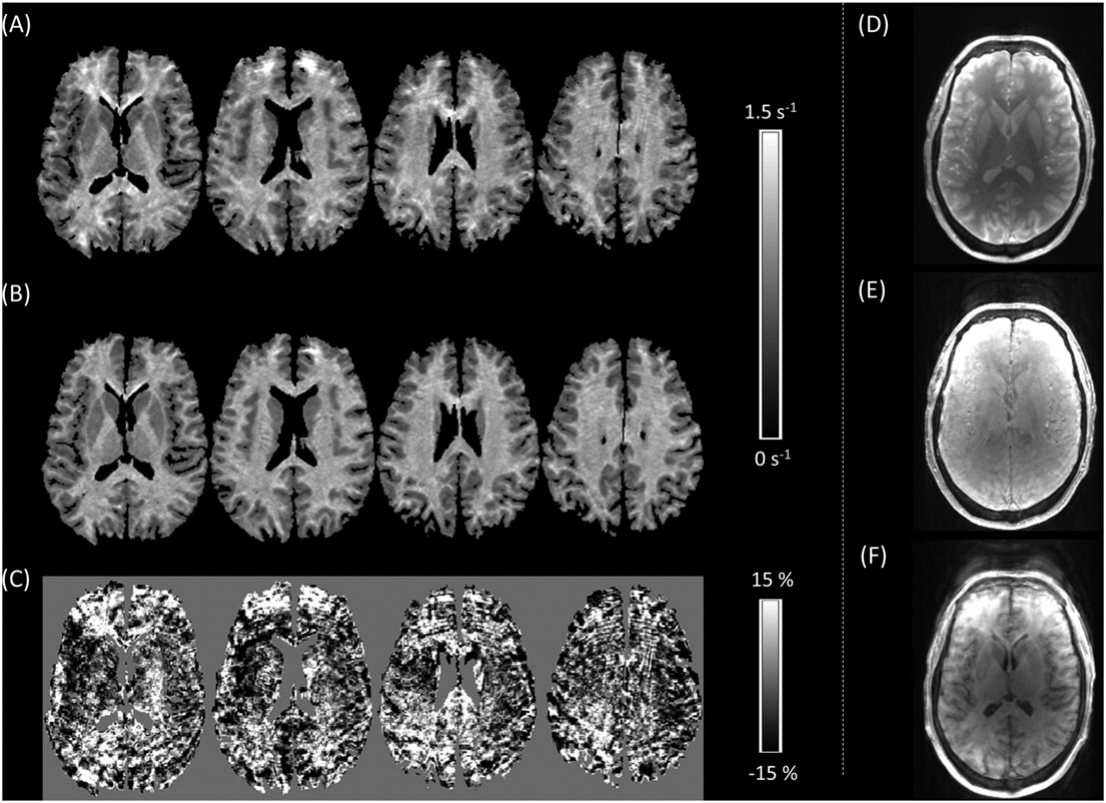
A, Multiple axial slices through the motion‐corrupted *R*_1_ map of Participant 2; B, the synthetic *R*_1_ map generated using the linear relaxometry model; C, the model residuals, expressed as the percentage of the mean of the measured and synthetic *R*_1_ values, which capture the motion artefact. D‐F, A single slice through the weighted volumes shows that the MT‐weighted acquisition D, is artefact free whereas the PD‐ E, and, to a greater extent, the *T*_1_‐weighted F, acquisitions are corrupted by motion artefacts

Results of the forced choice assessment are presented in Table 1. In 50% of cases, the perceived level of motion artefact was significantly reduced in one of the synthetic parameter maps. MT was improved in four cases. An example of an improved MT map is shown in Figure 1. *R*_1_ was improved in two cases and an example is shown in Figure 2. As would be expected, in no case was there a significant improvement in both synthesized maps.

**Table 1:** Percentage of five raters who selected a particular synthesized parameter map as having lower artefact levels. An asterisk indicates significance at *p* < 0.05

Participant	% raters selecting synthetic *R*_1_ map	% raters selecting synthetic MT map	Significant improvement
1	0*	100*	synthetic MT (Figure 1)
2	100*	20	synthetic *R*_1_ (Figure 2)
3	0*	100*	synthetic MT
4	20	40	—
5	80	20	—
6	20	100*	synthetic MT
7	80	0*	—
8	40	80	—
9	0*	100*	synthetic MT
10	100*	0*	synthetic *R*_1_
11	60	0*	—
12	20	80	—

### 4.2 GM segmentation

Improved segmentation performance, i.e. segmentation specificity, is expected to result in significantly higher GM probability in GM regions and/or significantly lower GM probability in WM regions.

#### 4.2.1 Synthetic MT maps compared with *T*_1_‐weighted data

**Figure 3:**
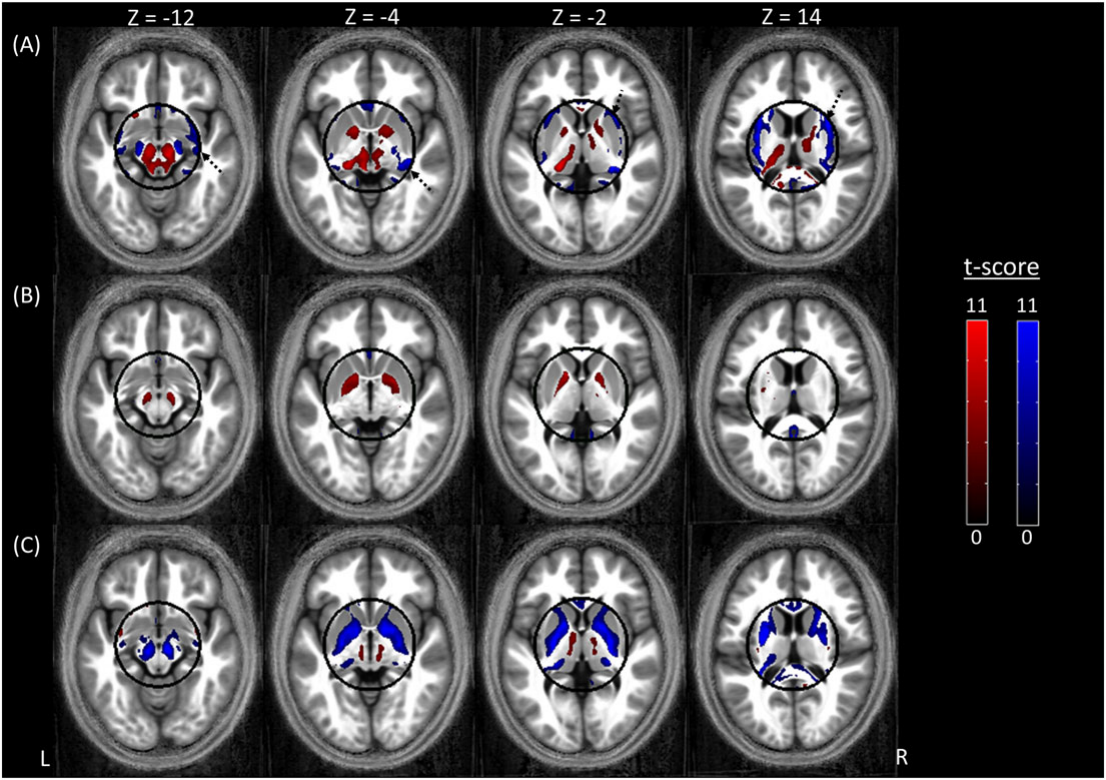
Regions showing either higher (red) or lower (blue) GM probability when using the synthetic quantitative MT map as input to the segmentation algorithm as compared with A, a *T*_1_‐weighted image, B, a quantitative *R*_1_ map, and C, a quantitative MT map. For display purposes only, the results are presented at a statistical threshold of *p* < 0.001 without correcting for multiple comparisons and are overlaid on the average normalized MT map of the cohort. Note that the statistical analysis was restricted to a sphere, with a radius of 4 cm, in the centre of the brain. The outline of the sphere is indicated in black. The dashed arrows in A indicate WM regions in which the GM probability was lower for the synthetic MT map

Table 2 lists significant differences between the segmentation of the synthetic MT maps and the *T*_1_‐weighted data. The synthetic MT map had significantly higher GM probability than the *T*_1_‐weighted data in the following GM regions: pulvinar nucleus of the left thalamus, left pallidum, within the brainstem, particularly the left and right substantia nigra, and in the pons at the level of the pontine reticular formation (Figure 3A, red; Table 2). The GM probability was significantly lower (Figure 3A, blue; Table 2) in one GM region (the left gyrus rectus), and in distributed bilateral WM regions encapsulated by the ROI (dashed black arrows in Figure 3A).

**Table 2:** Clusters in which the GM probabilities derived from the synthetic MT maps and *T*_1_‐weighted images were significantly different, *p* < 0.05 after small volume and family‐wise error correction. Clusters with fewer than 10 voxels were excluded

	Primary location	*p* value	Cluster extent	Peak *t* score	MNI coordinates		
					*x* [mm]	*y* [mm]	*z* [mm]
Synthetic MT > *T*_1_ weighted	Left thalamus and substantia nigra	<0.001	2445	11.18	−18	−28	−2
	Right substantia nigra	<0.001	675	10.78	8	−19	−17
	WM	<0.001	41	8.67	−19	16	−11
	Left substantia nigra	<0.001	27	7.69	−13	−22	−10
	Brainstem	<0.001	14	7.65	14	−27	−24
	Left pallidum	<0.001	37	7.28	−13	2	−3
	Pons	<0.001	10	7.15	1	−23	−33
Synthetic MT < *T*_1_ weighted	WM	<0.001	3258	15.30	28	−21	19
	Left gyrus rectus	<0.001	119	13.84	−1	16	−22
	WM	<0.001	2891	13.49	−34	−23	23
	WM lateral to left substantia nigra	<0.001	128	11.03	−15	−17	−13
	WM lateral to right substantia nigra; right thalamus	<0.001	164	10.56	17	−16	−12
	WM	<0.001	54	10.13	−37	−28	7
	WM	<0.001	57	9.07	32	3	−12
	Splenium	<0.001	30	8.87	19	−46	14
	WM	<0.001	130	8.61	−6	26	−1
	WM	<0.001	50	7.92	−7	20	23

#### 4.2.2 Synthetic MT maps compared with *R*_1_ maps

Table 3 lists significant differences between the segmentation of the synthetic MT and *R*_1_ maps. The synthetic MT map had significantly higher GM probability than the *R*_1_ map in the following GM regions: left and right pallidum and focally within the right substantia nigra (Figure 3B, red; Table 3). The GM probability was significantly lower in two GM regions: the left gyrus rectus, the right lingual gyrus and within the interhemispheric fissure (Figure 3B, blue; Table 3).

**Table 3:** Clusters in which the GM probabilities derived from the synthetic MT maps and *R*_1_ maps were significantly different, *p* < 0.05 after small volume and family‐wise error correction. Clusters with fewer than 10 voxels were excluded

	Primary location	*p* value	Cluster extent	Peak *t* score	MNI coordinates		
					*x* [mm]	*y* [mm]	*z* [mm]
Synthetic MT > *R*_1_ map	Left pallidum	<0.001	169	8.58	−13	5	2
	Right pallidum	<0.001	62	7.38	20	−1	−3
	Left pallidum	0.002	12	7.11	−23	−12	−3
	Right substantia nigra	0.002	10	6.76	13	−16	−12
Synthetic MT < *R*_1_ map	Left gyrus rectus	<0.001	210	11.40	−3	14	−24
	Interhemispheric fissure	<0.001	287	10.46	0	−49	17
	Right lingual gyrus	0.002	12	7.31	8	−47	2

#### 4.2.3 Synthetic MT maps compared with original measured MT maps

Table 4 lists significant differences between the segmentation of the synthetic and original MT maps. The synthetic MT map had significantly lower GM probability than the MT map in distributed GM regions encompassing the left and right pallidum, extending into the right putamen, in the left and right substantia nigra, in the gyrus rectus and in multiple WM regions (Figure 3C, blue; Table 4). The GM probability of the synthetic MT map was significantly higher in one WM voxel (Figure 3C, red).

**Table 4:** Clusters in which the GM probabilities derived from the synthetic MT maps and the original MT maps were significantly different, *p* < 0.05 after small volume and family‐wise error correction. Clusters with fewer than 10 voxels were excluded

	Primary location	*p* value	Cluster extent	Peak *t* score	MNI coordinates		
					*x* [mm]	*y* [mm]	*z* [mm]
Synthetic MT < MT map	Left pallidum	<0.001	3914	15.24	−22	−7	1
	Right pallidum	<0.001	4253	14.40	25	−13	−1
	WM	<0.001	534	13.98	−25	−33	19
	Right substantia nigra	<0.001	551	13.65	9	−18	−12
	Left gyrus rectus	<0.001	199	12.22	−2	14	−24
	Left substantia nigra	<0.001	491	12.21	‐9	−18	−11
	WM	<0.001	44	9.96	9	−32	19
	Left thalamus	<0.001	222	9.62	−21	−29	2
	Right thalamus	<0.001	63	9.50	23	−27	2
	Corpus callosum body	<0.001	44	8.99	4	−6	25
	WM	<0.001	105	8.92	23	−5	24
	WM	<0.001	33	8.90	26	−32	22
	Genu	<0.001	14	8.30	−3	22	10
	Corpus callosum body	<0.001	40	8.24	−2	−15	24
	Corpus callosum body	<0.001	17	8.01	−2	15	17
	Corpus callosum body	<0.001	12	7.69	−2	−25	22
	WM	<0.001	39	7.62	−20	−16	20

## 5 DISCUSSION

The MPM protocol produces maps of *R*_1_, *R*_2_* and MT. The interdependence of these maps is described, to a large extent, by the principled linear relaxometry model of *R*_1_. We have demonstrated how this model, together with population‐derived model coefficients, can be leveraged to *synthesize* quantitative maps from a subset of the MPM maps. This raises the possibility of generating synthetic quantitative maps of MRI parameters without actually acquiring the data typically required and introduces speed and/or robustness to quantitative imaging that will be of great importance in translating such approaches to a clinical environment.

Motion leads to inconsistencies across the imaging data used in the linear relaxometry model, which are largely captured by the model residuals facilitating artefact correction. Although motion is captured in the residuals these cannot be used to quantify the performance of the proposed method, since they will be influenced not only by artefact but also by any unspecified contributions to the measured *R*_1_, as well as any systematic bias. Therefore, to quantify the performance of the method we have used expert image quality rating, which is model independent and thus allowed an independent assessment of the method. This evaluation found significant data quality improvement in 50% of the cases evaluated by expert raters skilled in the identification of motion artefact.

In each case, significant artefact reduction was only achieved in one or other of the synthetic maps since at least a subset of artefact‐free maps are required to afford an improvement. Significant image quality improvements occurred most frequently for MT maps. Intra‐scan motion occurring only during the MT‐weighted acquisition will only affect the MT map. This is the optimal scenario for the presented correction scheme, an example of which is shown in Figure 1. Improvement occurred less frequently for synthesized *R*_1_ maps since both the PD‐ and *T*_1_‐weighted FLASH volumes used to calculate the *R*_1_ map will also contribute to the *R*_2_* and MT maps, and therefore if intra‐scan motion has occurred during the acquisition of these volumes artefact will be present in multiple maps, limiting the performance of the correction scheme, e.g. in Figure 2. In those cases for which no improvement was achieved, motion artefact was present to some extent in all of the constituent FLASH volumes and therefore all of the MPM maps.

In addition to variable performance, bias can be expected in the synthetic quantitative maps. We have previously reported^11^ that this bias can lead to reduced *R*_1_ in WM (of order 1.62%) and increased *R*_1_ in GM (of order 0.97%). This reduces the contrast between GM and WM, which is an important consideration if a parameter map is to be replaced with its motion‐artefact‐free synthetic counterpart. This bias can arise because of components that have not been explicitly included in the model. However, it has previously been shown that macromolecular and iron components, which are rather well captured by MT and *R*_2_* respectively,^31^, ^32^ are the dominant contributors to *R*_1_.^11^, ^15^, ^16^, ^33^, ^34^, ^35^ Any orientation‐dependent or higher‐order relationships that exist between the qMRI maps are not captured by the model either, and may also be a source of bias.^36^

Any relaxometry model, such as the one used in this work, must make simplifying assumptions about the nature of the interaction between water compartments. A central assumption concerns the timescale over which the interaction between the different water compartments of the tissue occurs, which may be short, intermediate or long.^14^ In our case, we build upon the assumption of fast exchange whereby we assume that the rate of exchange between compartments is higher than the difference in the relaxation rates of these constituent compartments.^37^ As a consequence, the signal we measure is a weighted sum of the constituent compartments visible via MRI.^11^, ^14^ There are multiple reports that support this assumption by showing that the longitudinal relaxation rate in the brain is well described by a mono‐exponential form and that deviations from this behaviour are small.^e.g.^ 15, 38 Limitations in the validity of this assumption may be a source of bias. Models such as the one presented here are also a first step towards *in vivo* histology,^2^ in which quantitative maps are combined using biophysical models in order to extract descriptors of the underlying tissue such as myelin and iron levels^3^ or the degree of myelination of fibres.^48^ Any biases present in the parameter maps derived from this model would propagate through to these biological descriptors. Further development of the model will allow us to test and refine the validity of the model assumptions, such as the exchange between water compartments within the brain. Future work will assess if it is possible to achieve the desired motion artefact correction without introducing bias, e.g. through the use of regularization and the inclusion of higher‐order effects determining the relationship between the underlying features of the tissue microstructure, quantitative parameter maps and image contrast.

Clearly there are many other approaches to motion artefact correction, e.g. optical motion tracking,^39^ estimating motion directly from the MR signal itself^40^, ^41^, ^42^ or retrospective approaches that operate on the acquired *k*‐space data.^43^ However, these approaches have the respective drawbacks of requiring additional hardware, sequence modification and associated time‐penalties, or significant computational effort. The approach presented here follows the idea of maximally exploiting consistencies across multiple data sets acquired as part of a single quantitative imaging protocol, e.g. as done in the ESTATICS approach to creating maps of the effective transverse relaxation rate from multiple image contrasts.^44^

An MT‐weighted image is required in order to calculate a map of the percentage saturation due to MT or similar measures such as the MTR.^45^ If an MT‐weighted image is not available, e.g. because of insufficient scan time or because motion has corrupted the data, we have shown that synthetic maps of MT can nonetheless be generated using the linear relaxometry model with population‐derived model coefficients. When the GM probabilities derived from this synthetic MT map were compared with those derived from *T*_1_‐weighted data we found that the synthetic MT map had significantly higher GM probability in deep GM structures such as the substantia nigra, at the interface between the caudate and thalamus and in the anterior portion of the pallidum. Improved performance was also found for the synthetic MT maps in the substantia nigra and pallidum when compared with quantitative *R*_1_ maps. Additionally, both comparisons only found significantly lower GM probabilities in WM regions, i.e. the synthetic MT map more accurately classified the different tissue types. It is not surprising that the synthetic MT map did not outperform the original MT map. This finding points towards some limitations of the relaxometry model, for example the issue of bias discussed previously.

Biophysical models can provide insights into morphometric studies that show differential GM volume estimation, which is dependent on the contrast of the data used as input to the segmentation routine used for the morphometry.^12^, ^46^ Understanding these effects is of critical importance for computational neuroanatomy studies, particularly when there is ambiguity over the origin of observed changes, e.g. due to co‐localized and interacting effects of atrophy and MRI parameter changes, as occur during ageing.^46^ It has also been shown that age‐related atrophy and differences in tissue microstructure can be captured by the qMRI parameters used in this study.^30^, ^46^ To circumvent any atrophy‐related confound being introduced here, the cohort used was restricted to a narrow age range (18–25 years).

The linear relaxometry model itself assists with the interpretation of the differential segmentation performance we have seen. Considering the pallidum for example, the effects of reduced myelin content and increased iron content counteract each other in the *R*_1_ map whereas in the synthetic MT map the effect of reduced myelin content in the pallidum should dominate since the iron effect has largely been removed. For this reason, there is greater contrast between the pallidum and the surrounding WM in the synthetic MT map than in the *R*_1_ map, resulting in improved delineation of GM and WM by the segmentation algorithm. These effects of iron and myelin also combine to increase contrast in the *T*_1_‐weighted acquisition. The reduced myelin content of the pallidum means that the *T*_1_ relaxation time is longer than in the surrounding WM, leading to reduced signal intensity on a *T*_1_‐weighted acquisition. The inevitable *T*_2_* weighting that is also present, since a *T*_E_ of 0 ms cannot be achieved, means that the higher iron content additionally reduces the signal intensity of the acquisition due to more rapid signal decay. Hence, rather good segmentation performance was achieved for this structure using the *T*_1_‐weighted acquisition as input.

In keeping with previous studies comparing segmentation performance on *T*_1_‐weighted MDEFT (modified driven equilibrium Fourier transform)^47^ and MT maps,^12^ we also find that the MT map is the optimal choice to drive the segmentation algorithm, since it supports an even better delineation of subcortical structures (Figure 3). Therefore, if the time is available to acquire the extra data required to calculate a map of MT saturation, and if it is not corrupted by motion artefact, then this quantitative map should be the first choice for morphological studies based on segmentation. It should be borne in mind that the segmentation performance will also depend on the algorithm and prior information, i.e. the TPMs, used. These were constant across all analyses presented here, which utilized the segmentation algorithm and default TPMs of SPM12.

While this demonstration has been specific to the MPM protocol, the approach could be extended to other protocols to generate synthetic quantitative maps of MRI parameters without acquiring the data typically required. This raises the possibilities of improving efficiency and/or robustness in quantitative imaging, which is of great importance in translating such approaches to a clinical environment.

## 6 CONCLUSIONS

Modelling and exploiting the interdependence of quantitative parameter maps facilitates greater insights into the underlying tissue microstructure and the removal of artefactual inconsistencies, e.g. due to head motion. Robustness to motion is a key requirement for quantitative imaging, particularly for the study of non‐compliant participants, such as patients suffering from movement disorders. Here we have shown improved robustness to motion by creating synthetic qMRI maps free of artefact by applying biophysical models to motion‐corrupted data. In addition, synthetic MT maps have been used to demonstrate improved segmentation of deep GM structures in comparison to that achieved with conventional *T*_1_‐weighted data or quantitative *R*_1_ maps. The proposed synthetic qMRI approach shows promise for furthering our understanding of the inter‐relation of MRI parameters and for maximizing the extraction from qMRI protocols of high quality *in vivo* histology information related to tissue microstructure.
